# GM-CSF-dependent CD301b+ lung dendritic cells confer tolerance to inhaled allergens

**DOI:** 10.21203/rs.3.rs-4414130/v1

**Published:** 2024-06-04

**Authors:** Christina L. Wilkinson, Keiko Nakano, Sara A. Grimm, Gregory S. Whitehead, Yukitomo Arao, Perry J. Blackshear, Peer W. Karmaus, Michael B. Fessler, Donald N. Cook, Hideki Nakano

**Affiliations:** 1 Immunity, Inflammation and Disease Laboratory, Division of Intramural Research, National Institute of Environmental Health Sciences, NIH, Research Triangle Park, North Carolina 27709, USA; 2 Integrative Bioinformatics Support Group, Division of Intramural Research, National Institute of Environmental Health Sciences, NIH, Research Triangle Park, North Carolina 27709, USA; 3 Signal Transduction Laboratory, Division of Intramural Research, National Institute of Environmental Health Sciences, NIH, Research Triangle Park, North Carolina 27709, USA

## Abstract

The severity of allergic asthma is driven by the balance between allergen-specific T regulatory (Treg) and T helper (Th)2 cells. However, it is unclear whether specific subsets of conventional dendritic cells (cDCs) promote the differentiation of these two T cell lineaeges. We have identified a subset of lung resident type 2 cDCs (cDC2s) that display high levels of CD301b and have potent Treg-inducing activity *ex vivo*. Single cell RNA sequencing and adoptive transfer experiments show that during allergic sensitization, many CD301b^+^ cDC2s transition in a stepwise manner to CD200+ cDC2s that selectively promote Th2 differentiation. GM-CSF augments the development and maintenance of CD301b^+^ cDC2s *in vivo*, and also selectively expands Treg-inducing CD301b^+^ cDC2s derived from bone marrow. Upon their adoptive transfer to recipient mice, lung-derived CD301b^+^ cDC2s confer immunological tolerance to inhaled allergens. Thus, GM-CSF maintains lung homeostasis by increasing numbers of Treg-inducing CD301b^+^ cDC2s.

## Introduction

Allergic asthma is a widespread disease characterized by reversible airway obstruction, inflammation, and airway hyper-responsiveness (AHR) ^1^. Many asthmatics have predominantly eosinophilic inflammation of the airway and have high levels of the type 2 cytokines, IL-4, IL-5 and IL-13, which promote IgE production, eosinophilic inflammation, mucus production and AHR. Allergen-specific T helper (Th)2 cells are a major source of these cytokines. During allergic sensitization, these cells develop from naïve CD4^+^ T cells that interact with conventional dendritic cells (cDCs) presenting allergen-derived peptides in the context of MHC class II (MHC-II). However, the presence of allergen-specific Th2 cells does not always lead to allergic disease because peripheral T cells in healthy individuals and in asthmatics recognize the same allergen-specific epitopes ^2^. One critical difference between asthmatics and individuals with healthy airways is that the former lack sufficiently strong regulatory mechanisms to hold allergen-specific effector responses in check ^3, 4^. Accordingly, there has been considerable interest in increasing the strength of allergen-specific regulatory responses through subcutaneous immunotherapy (SCIT) or sublingual immunotherapy (SLIT) with the provoking allergens ^5^. Although often effective, the allergen dosing must be continuously maintained for 3 years ^6^, and this has led to poor patient compliance and reduced efficacy of the therapy ^7^. Thus, there is an unmet need for immunotherapeutic strategies that have a shorter timeline and consequent improved patient compliance.

A wealth of evidence has shown that regulatory CD4^+^ T cells (Tregs) can strongly suppress allergic responses ^8^, including established inflammation of the airway ^9^. These cells are characterized by their cell surface display of CD25 (IL-2 receptor alpha subunit) and the Treg master transcription factor, Foxp3 ^10^. Several studies have shown that asthma patients have fewer CD25^+^Foxp3^+^ Tregs in the peripheral blood than non-asthmatic individuals ^11, 12^. Although TGF-β and IL-2 have been shown to promote Treg development *in vitro* ^13^, much less is known of how these cells arise *in vivo*. In particular, it is unclear whether a specific subset of lung cDCs promote allergen specific Treg development, or whether all lung cDCs have this capacity, depending on the environmental signals they receive. The identification of a dedicated Treg-inducing cDC subset should facilitate mechanistic studies of Treg development *in vivo* and might lead to improved DC-based immunotherapies for asthma.

The lungs of humans and mice contain two major cDC subsets, usually referred to as cDC1 and cDC2 ^14^. Both of these cDC subsets develop from FMS-like tyrosine kinase 3 ligand (FLT3L)-dependent DC precursors (preDCs) ^15^ and are therefore developmentally distinct from monocyte-derived macrophages that arise independently of FLT3L ^16, 17^. Mouse cDC1s are homogeneous and can be readily identified by their display of CD103 (αE integrin) and relatively low amounts of CD11b (αM integrin). By contrast, mouse cDC2s are very heterogeneous, have low amounts of CD103 and display high levels of CD11b. The heterogeneity of cDC2s has confounded efforts to assign specific functions to these cells, but the advent of single cell RNA-sequencing (scRNA-seq) has provided some much needed clarity ^18, 19, 20, 21^. Our group recently reported that the cell surface marker CD301b (encoded by *Mgl2*) is present on the cell surface of most lung cDC2s at steady state ^18^. However, during allergen/adjuvant-mediated allergic sensitization through the airway, additional subsets of cDC2s accumulate in the lung. One such subset displays Ly6C and potently stimulates Th17 differentiation, whereas a different subset has high display levels of CD200 and drives Th2 differentiation ^18^.

In the present report, we studied lung cDCs that promote Treg differentiation and confer allergen-specific tolerance. We found that CD301b^+^ cDC2s strongly promote the development of allergen-specific CD25^+^Foxp3^+^ Tregs. However, during house dust extract (HDE)-mediated allergic sensitization, many CD301b^+^ cDC2s transition to more mature Th2-inducing cDC2s. The number of Treg-inducing cDCs is controlled in part by the cytokine granulocyte-macrophage colony-stimulating factor (GM-CSF) because mice lacking *Csf2rb* in DCs have fewer CD301b^+^ cDC2s and more Th2-inducing cDC2s. CD301b^+^ cDC2s display low levels of costimulatory molecules and express high levels of *Tgfb1*. Inhibition of signaling from TGF-β receptor suppresses Treg induced by cDC2s. Taken together, these findings show that GM-CSF maintains immunotolerance in the lung by increasing numbers of Treg-inducing cDC2s.

## Results

### Lung CD301b^+^ cDC2s induce Treg differentiation *ex vivo* and immunotolerance *in vivo*

**Fig. 1 |:**
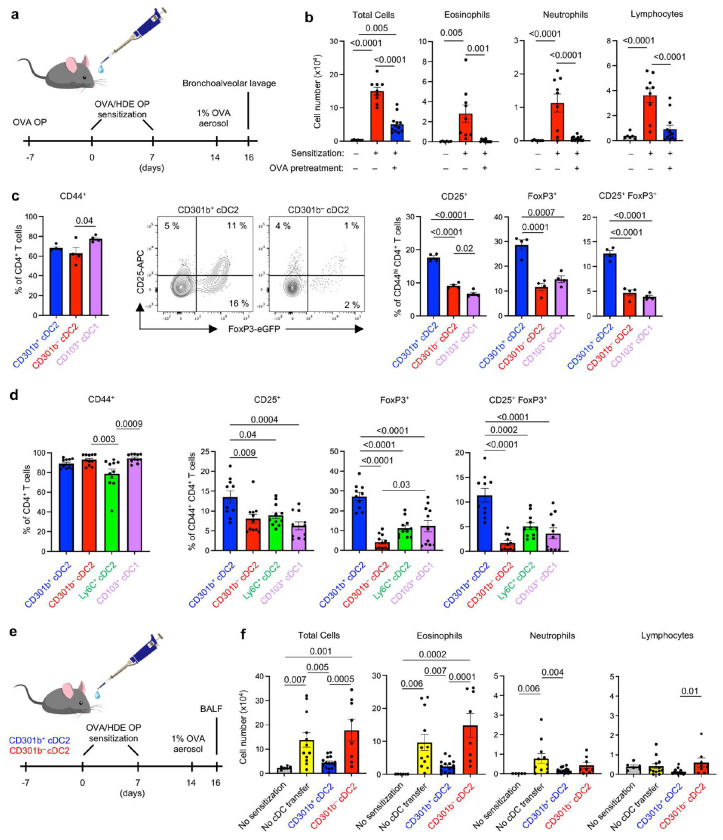
CD301b^+^ lung cDC2s promote Treg differentiation and induce immunological tolerance. **a**, Timelines for mouse models of asthma. To induce asthma, mice were sensitized with OVA/HDE twice by o.p. and challenged once with OVA aerosol. Bronchoalveolar lavage fluid (BALF) was harvested 48 h post- challenge. Assays of tolerance assays were similar, except that mice were also exposed to OVA alone by o.p. aspiration on day −7. **b**, Cell numbers for the indicated leukocytes in BALF, as determined by differential microscopy (*n*=8-12). **c**, Activation of CD4^+^ T cells (left), representative cytograms of CD4^+^CD44^hi^ T cells (middle) and compiled data of Tregs (right) are shown. Naïve CD4^+^ T cells from *Foxp3^eGFP^* OT-II mice were cocultured for 5 days with indicated cDC2 subsets isolated from C57BL/6 mice that received OVA by o.p. aspiration (*n*=4). Gating strategy for purified cDC subsets and CD4^+^ T cell analysis (CD4^+^CD3ε^+^MHCII^−^Live/Dead^−^) is shown in Extended Data Fig. 1a and b. **d**, Activation of CD4^+^ T cells (left) and Treg induction in CD44^+^ CD4^+^ T cells (right) by distinct cDC subsets isolated from mice that received OVA/HDE by o.p. aspiration (*n*=5). **e**, Timeline for mouse model of asthma to test tolerogenic function of cDC2 subsets. CD301b^+^ or CD301b^−^ cDC2s were purified and incubated with OVA_323-339_ peptides, then adoptively transferred by o.p. to C57BL/6 mice on day 0. After OVA/HDE sensitization and OVA challenge, cells in BALF were analyzed. **f**, Cell numbers of the indicated leukocytes in BALF. (*n*=6-16). (**b**, **c**, **d**) Data were analyzed by one-way ANOVA with Tukey’s multiple comparison test. (**f**) Data were analyzed by one-way ANOVA with LSD test. Data are presented as mean values ± SEM. *P* values are indicated above the graphs. (**b**, **f**) Each dot represents individual mouse. Combined results from two independent experiments are shown. (**c**, **d**) Each dot represents separately cultured CD4^+^ T cells. Representative results from 2 independent experiments are shown.

It has long been known that mice become immunotolerant to the experimental allergen, ovalbumin (OVA), upon inhaling aerosols of that protein ^22^. We confirmed this, and further showed that mice receiving oropharyngeal (o.p.) instillations of highly purified OVA prior to allergic sensitization also become tolerant to OVA (Fig. 1a). Thus, although mice that received o.p. instillations of HDE mixed with OVA became sensitized to OVA and developed robust allergic airway inflammation upon subsequent OVA challenge, mice that had received o.p. instillations of purified OVA prior to HDE/OVA sensitization had clearly diminished responses to OVA challenge (Fig. 1b). In particular, eosinophil and neutrophil inflammation were virtually abolished by pre-treatment with purified OVA prior to OVA/HDE sensitization. We therefore concluded that this model of allergen-specific tolerance would be appropriate for studying tolerogenic cDCs in the lung.

To determine whether a specific cDC subset preferentially induces Treg differentiation, we treated mice with tolerance-inducing, purified OVA and used antibodies against the subset-specific cell surface markers to purify the various cDC2 subsets by flow cytometry. Total cDCs were identified as CD11c^+^MHCII^+^CD88^−^F4/80^−^SiglecF^−^, thus excluding alveolar macrophages (SiglecF^+^CD88^+^F4/80^+^), interstitial macrophages (CD88^+^F4/80^+^), monocytes (F4/80^+^) and neutrophils (CD88^+^) (Extended Data Fig. 1a). Within this total cDC population, cDC1s were identified as CD103^+^CD11b^lo^ and cDC2s as CD103^−^CD11b^hi^. cDC2s were further stratified to CD301b^+^ cDC2s and CD301b^−^ cDC2s. The two cDC2 populations, as well as cDC1s, were cultured *ex vivo* separately with naïve CD4^+^ T cells isolated from OT-II OVA-specific T cell receptor transgenic mice bearing a *Foxp3^eGFP^* reporter gene to allow Treg detection by flow cytometry (Extended Data Fig. 1b). After 5 days of coculture, CD103^+^ cDC1s, as well as CD301b^+^ and CD30lb^−^ cDC2s, had activated OVA-specific CD4^+^ T cells, as indicated by the increased display of the activation marker CD44. Treg cells, identified by their display of CD25 and GFP fluorescence encoded by the *Foxp3^eGFP^* gene, were more efficiently induced by CD301b^+^ cDC2s than by any of the other cDC subsets (Fig. 1c). These data show that at least under tolerogenic conditions, CD301b^+^ lung cDC2s strongly promote Treg induction *ex vivo*.

Although instillation of highly purified OVA efficiently promotes immunotolerance, most allergen preparations are contaminated with adjuvants in the form of bacterial products such as LPS and flagellin ^23^. These products, as well as HDE, possess adjuvant activity and can promote allergic sensitization to co-inhaled OVA ^23, 24^. However, Treg induction and tolerogenic responses to inhaled allergens can occur even in the presence of adjuvants ^25, 26^. The profile of cDC2 subsets in the lung changes during allergic sensitization, as Ly6C^+^ inflammatory cDC2s are recruited to the lung and CD200^+^ cDC2s undergo expansion ^18^. It remained possible, therefore, that the cDC subset(s) that drive regulatory responses during adjuvant-mediated allergic sensitization are different from the CD301b^+^ cDC2 subset that promotes Treg differentiation at steady state. To test this, we purified cDC subsets from lungs of mice that had been sensitized by o.p. instillations of OVA/HDE, and cocultured these cells with naïve CD4^+^ T cells from *Foxp3^eGFP^* OT-II reporter mice. CD301b^+^ cDC2s promoted significantly more CD25^+^ and Foxp3^eGFP^ Tregs compared with all other subsets, including Ly6C^+^ cDC2s (Fig. 1d). The latter promoted Th17 cells, whereas CD200^+^ cDC2s predominantly promoted Th2 differentiation in agreement with a previous report ^18^ (Extended Data Fig. 1c and d). Taken together, these results demonstrate that individual cDC2 subsets in the lung have unique functions and that CD301b^+^ cDC2s promote Treg differentiation not only at steady state, but also during allergic sensitization.

The unique ability of cDCs to activate naïve T cells has been effectively harnessed in DC-based vaccines. The best-known examples of this are therapies in which tumor antigen-bearing DCs are injected into cancer patients to strengthen their anti-tumor immunity ^27^. In allergic diseases such as asthma, the opposite effect is desired, namely, to diminish the strength of immune responses to provoking allergens. To test the feasibility of using DC-based therapy to confer tolerance to allergens *in vivo*, we purified CD301b^+^ or CD301b^−^ cDC2s from naïve wildtype (WT) mice, incubated these cells with OVA_323-339_ peptide and adoptively transferred the cells to naïve mice by o.p. instillation. One week later, the mice were subjected to the OVA/HDE model of allergic asthma and their cellular inflammation in airways evaluated (Fig. 1e). Mice that had received OVA-loaded CD301b^+^ cDC2s prior to allergic sensitization had significantly fewer inflammatory cells, including eosinophils and neutrophils, in the airway after challenge compared with mice that did not receive the cDC pretreatment (Fig. 1f). By contrast, the inflammation in mice that received OVA-loaded CD301b^−^ cDC2s was comparable to that of animals that did not receive cDC pretreatment. Thus, adoptive transfer of allergen-loaded CD301b^+^ cDC2s specifically confers tolerance to inhaled allergens *in vivo*.

### Lung resident CD301b^+^ cDC2s transition to Th2-inducing cDCs during allergic sensitization

**Fig. 2 |:**
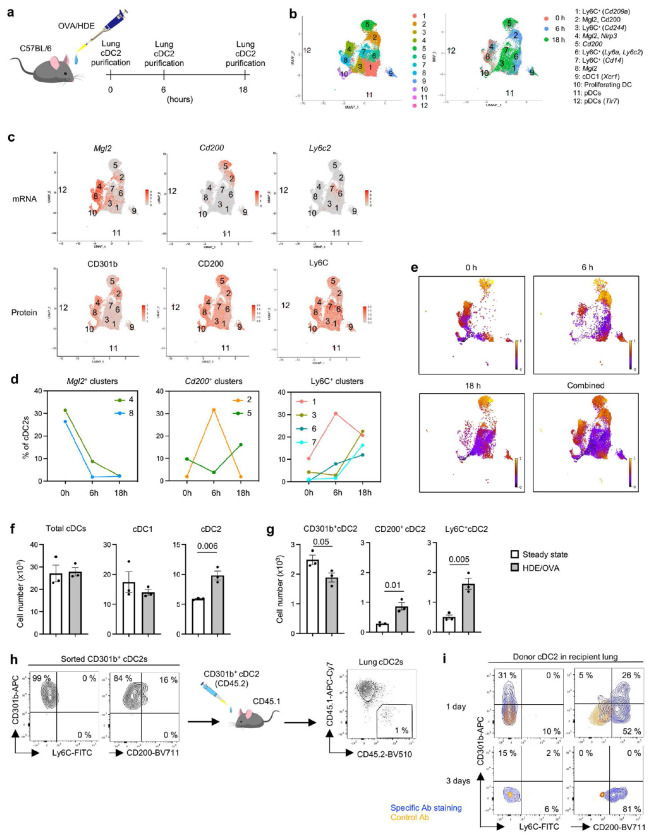
CD301b^+^ lung resident cDC2s give rise to CD200^+^ migratory cDC2s. **a**, Timeline for lung cDC2 isolation following OVA/HDE sensitization. Lung cells were harvested at 0 h (steady state), 6h, and 18h post-sensitization, and purified cDC2 were analyzed by CITE-Seq. **b**, UMAP plots showing cDC2 clusters identified in scRNA-Seq (left panel). Clusters are denoted by collection timepoints (right panel). **c**, UMAPs showing expression of subset-marker genes (top) and proteins (bottom). **d**, Time course of each cluster’s abundance in cDC2s at steady state and after allergic sensitization. **e**, RNA velocity analysis of cDC2s at each time point and combination. Maturation stages inferred by scVelocity latent time are indicated by different colors. **f**, **g**, The abundance of lung cDCs at steady state and 16 h after sensitization with OVA/HDE were calculated based on total cell counts and flow cytometric analysis. Each dot represents an individual mouse. Data were analyzed by two-tailed t-test (*n*=3) and presented as mean values ± SEM. *P* values are indicated above the graphs. **h**, Adoptive transfer of purified CD301b^+^ lung cDC2s from C57BL/6 mice (CD45.2) to CD45.1 recipient mice. Representative cytograms of purified donor cells and recipient lung cDC2s post-transfer are shown. **i**, Lung cDC2s at 1 day and 3 days post-transfer were analyzed by flow cytometry. Cytogram showing the phenotype of donor CD301b^+^ cDC2s-derived cells at the indicated time points. (**a**, **f**, **g**, **h**) Gating strategies depicted in Extended Data Figures 3a-c. (**f**, **g**, **h**, **i**) Representative results from 2 independent experiments are shown.

To better understand the relationships between Treg-inducing CD301b^+^ cDC2s and other cDC2 subsets in the lung, we employed scRNA-seq with cellular indexing of transcriptomes and epitopes (CITE-Seq). Mice were harvested at 0h (baseline), 6h, and 18h post-allergic sensitization with OVA/HDE, and total CD11b^+^ lung cDC2s were prepared and analyzed (Fig. 2a, Extended Data Fig. 2a). Cells from the three preparations were pooled and sequenced simultaneously, but different barcodes were used for each time point, allowing us to assign a specific time point to each cell during the analysis. Uniform Manifold Approximation and Projection (UMAP) revealed 12 clusters including 9 cDC2 clusters (Fig. 2b). *Mgl2*-expressing cells and protein CD301b^+^ cells in clusters 8 and 4 were the predominant cDC2s at baseline and were therefore designated ‘lung resident’ cDC2s (Fig. 2c and d). Clusters 2 and 5 did not become major clusters until 6h and 18h post-sensitization, respectively, with cells in both clusters expressing *Cd200*, as well as the *Ccr7* chemokine receptor gene (Fig. 2c and d, Extended Data Fig. 2b). Clusters 1, 3, 6 and 7 also appeared post-sensitization and displayed Ly6C (Fig. 2c and d, Extended Data Fig. 2c).

We next analyzed the scRNA-Seq data by RNA velocity ^28^, which is used to infer maturation stages of cells based on relative amounts of spliced and unspliced RNA. This approach suggested that during OVA/HDE-mediated allergic sensitization, multiple differentiation events occur simultaneously. Thus, *Mgl2*-expressing lung resident cDC2s in cluster 4 give rise to *Cd200*-expressing cDC2s in cluster 2 (Fig. 2e, Extended Data Fig. 2d). The latter appear to be a transitional cell type linking CD301b^+^ cDC2s with the more mature CD200^+^ cDC2s in cluster 5 (Fig. 2c and d). By contrast, *Mgl2*-expressing lung resident cDC2s in a different cluster (cluster 8) transition to proliferating DCs in cluster 10 (Extended Data Fig. 3c). In parallel with these changes, newly recruited *Ly6c*-expressing cDC2s in cluster 7 transition to cluster 3, whereas cDC2s in cluster 6 transition to cluster 2 (Fig. 2e, Extended Data Fig. 2d). The latter observation suggests that during allergic sensitization, *Cd200*^+^ transitional cDC2s in cluster 2 can arise either from CD301b^+^ (*Mgl2*^+^) cDC2s or from newly recruited Ly6C^+^ cDC2s. Overall, allergic sensitization through the airway triggers an increase in Ly6C^+^ inflammatory cDC2s and CD200^+^ *Ccr7*^+^ migratory cDC2s, with a corresponding decline in lung resident CD301b^+^ cDC2s (Fig. 2f and g, Extended Data Fig. 3a). However, the Ly6C^+^ cDC2s that arise post-sensitization can also develop into CD301b^+^ cDC2s, suggesting a pathway for replenishing the lung resident cDC2 population and thus maintaining homeostasis.

To experimentally confirm the developmental trajectory suggested by the RNA velocity analysis, we performed DC adoptive transfers with specific cDC2 subsets. CD301b^+^ cDC2s were prepared from CD45.2 donor mice and transferred into CD45.1 recipient animals (Fig. 2h, Extended Data Fig. 3b). By one day post-transfer, all CD301b^+^ donor DCs had gained CD200 and many had lost CD301b (Fig. 2i). By 3 days post-transfer, all donor cells were negative for CD301b, indicating a complete conversion of donor CD301b^+^ cDC2s to CD200^+^ cDC2s.

We used similar adoptive transfer experiments to study the developmental potential of Ly6C^+^ donor cDC2s. At one day post-transfer, all Ly6C^+^ donor cDC2s had reduced display of Ly6C and increased display of CD200 (Extended Data Fig. 3c and d). These results suggest that the majority of lung cDC2s are in the same lineage, which is in agreement with a recent report demonstrating that the type B cDC2 (cDC2B) lineage is the dominant cDC2 population in the lung and that these cells descend from a common preDC2 progenitor ^21^.

### Tregs can be induced in the lung

**Fig. 3 |:**
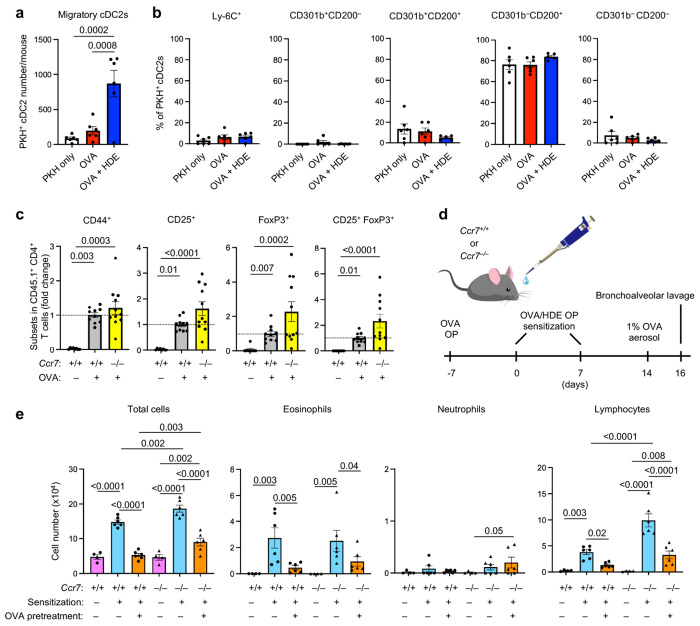
Tregs can be induced in the lung without cDC migration. **a**,**b**, Migration of cDC2 subsets. All C57BL/6 mice received PKH26 dye and some mice received OVA or OVA/HDE by o.p. aspiration. The frequency of each subset in PKH^+^ migratory cDC2s in mLNs of the recipient mice were evaluated by flow cytometry (*n*=5-6). Gating strategy depicted in Extended Data Figure 4a. Representative results of 2 independent experiments are shown. **c**, Treg generation in the lung of WT and *Ccr7*^−/−^ mice. All mice received naïve CD4^+^ T cells isolated from CD45.1 OT-II mice by intravenous injection, and OVA by o.p. aspiration. The phenotype of CD45.1^+^ donor-derived CD4^+^ T cells was analyzed by flow cytometric analysis of surface proteins and intracellular Foxp3 (*n*=10-11). Gating strategy depicted in Extended Data Figure 4b. **d**, Timeline for mouse model of asthma to test tolerance induction. Some WT and *Ccr7*^−/−^ mice received OVA by o.p. aspiration and were sensitized with HDE/OVA. After OVA aerosol challenge, cells in BAL fluid were analyzed. **e**, Cell numbers of the indicated leukocytes in BALF (*n*=4-6). (**a**, **b**, **c**, **e**) Each dot represents an individual mouse. Data are presented as mean values ± SEM. (**a**, **b**, **e**) Data were analyzed by one-way ANOVA with Fisher’s LSD test. (**c**) Data were analyzed by Kruskal-Wallis test. *P* values are indicated above the graphs.

The absence of *Ccr7* expression in CD301b^+^ cDC2s (Extended Data Fig. 2b) suggested that these cells are non-migratory. To test this, we labeled lung cDCs *in situ* by instilling the fluorescent dye, PKH, into the airways of mice. On the following day, lung-draining mediastinal lymph nodes (mLNs) were harvested and PKH^+^ migratory cDCs were identified by flow cytometry. Although a small number of migratory cDCs were detected in mLNs following treatment of mice with OVA alone, these cells were markedly increased in animals that had been sensitized with OVA/HDE (Fig. 3a). Furthermore, under all conditions tested, the vast majority of migratory cells in mLNs were CD200^+^, with very few CD301b^+^ cDC2s detected (Fig. 3b, Extended Data Fig. 4a). These results are consistent with a previous report ^18^ and confirm that CD301b^+^ cDC2s are lung resident, non-migratory cells. The virtual absence of *Ccr7* expression in lung resident CD301b^+^ cDC2s led us to test whether immunological tolerance can occur in the absence of cDC migration to mLNs. To this end, we tested Treg induction in *Ccr7*^−/−^mice, whose cDCs cannot migrate to regional LNs.^29^ naïve CD4^+^ T cells from CD45.1 OT-II mice were adoptively transferred into WT and *Ccr7*^−/−^mice, and numbers of FoxP3^+^ and CD25^+^ Tregs within the CD45.1 donor cell gate were evaluated following treatment of the recipient animals with OVA alone. As expected, development of CD44^hi^ effector CD4^+^ T cells and Tregs in the lungs of WT mice was dependent on OVA treatment (Fig. 3c, Extended Data Fig. 4b). The frequency of OVA-specific CD45.1 Tregs in *Ccr7*^−/−^ mouse lungs was as high, or higher, than that seen in WT mice, suggesting that Tregs can be generated in the lung in the absence of cDC migration. These results prompted us to further test whether allergen-specific immunological tolerance can also be induced without cDC migration by exposing *Ccr7*^−/−^ mice to OVA prior to allergic sensitization (Fig. 3d). Pretreatment with OVA by o.p. significantly reduced inflammatory cell accumulation in both WT and *Ccr7*^−/−^ compared with mice that were sensitized and challenged without pretreatment (Fig. 3e), although inflammatory cells were slightly more abundant in tolerance-induced *Ccr7*^−/−^ mice than in their WT counterparts. Together, these results suggest that Treg induction and immunological tolerance can be induced by lung resident cDCs.

### CD301b^+^ cDC2 development is promoted by GM-CSF

**Fig. 4 |:**
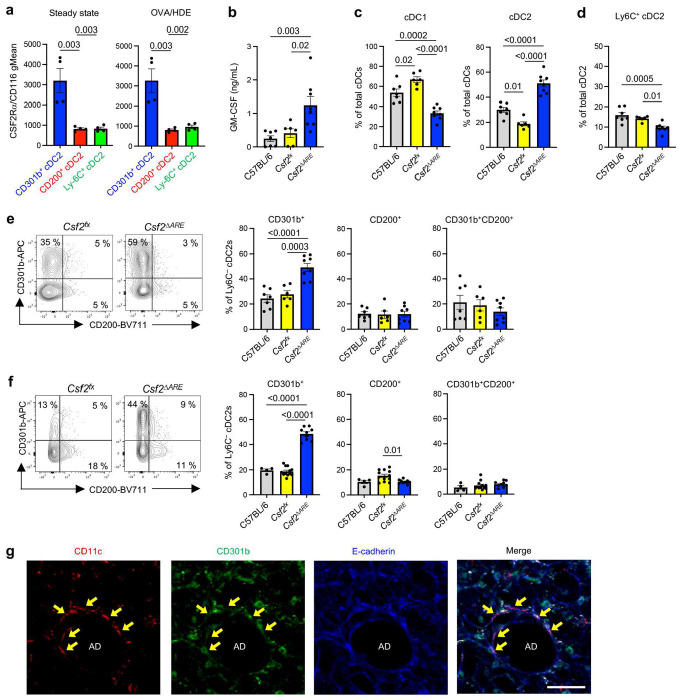
GM-CSF promotes the development of lung CD301b^+^ cDC2s. **a**, Surface display level of CSF2Rα on lung cDC2 subsets at steady state and 16 h after OVA/HDE inhalation were analyzed by flow cytometry. **b**, GM-CSF level in lung homogenates was measured by ELISA (*n*=5-7). **c-f**, The frequencies of cDC1s and cDC2 subsets in mouse lungs were analyzed by flow cytometry at steady state (**c-e**) or after OVA/HDE inhalation (**f**). (**e**, **f**) Representative cytograms (left panels) and compiled data (right panels) of Ly6C^−^ cDC2s are shown (*n*=6-8). Gating strategy depicted in Extended Data Fig. 6a. (**g**) Representative image of an alveolar duct in a PCLS of a mouse lung analyzed by a laser-scanning microscope. Individual staining for CD11c (red), CD301b (green), and E-cadherin (blue) are shown, as well as a merged image. Arrows indicate CD11c^+^CD301b^+^ cells. AD indicates alveolar duct. Scale bar represents 50 μm. (**a-f**) Data are presented as mean values ± SEM. Each dot represents an individual mouse. Data were analyzed by one-way ANOVA with Tukey’s multiple comparison test. Combined results from two independent experiments are shown.

The majority of cDCs, including cDC2s, depend on the growth factor FLT3L for their development ^15^. In addition, previous studies have revealed that GM-CSF is also required for homeostasis of cDC1s and cDC2s in multiple tissues, including lung ^30^. However, it is unknown whether some cDC2 subsets are particularly responsive to GM-CSF and if that cytokine impacts the relative abundance of different cDC2 subsets. RNA-Seq and scRNA-Seq analyses verified that bone marrow preDCs and mature lung cDCs express the *Csf2* receptor genes, *Csf2ra, Csf2rb*, and *Csf2rb2* (Extended Data Fig. 5a and b). Interestingly, flow cytometric analysis revealed that CD301b^+^ cDC2s display significantly higher levels of CSF2Rα protein than did Ly6C^+^ or CD200^+^ cDC2s either at steady state or after allergic sensitization (Fig. 4a).

Given the high display levels of the GM-CSF receptor on CD301b^+^ cDCs, we studied the *in vivo* effect of GM-CSF on those cells. To test whether overproduction of GM-CSF affects *in vivo* numbers of CD301b^+^ lung cDC2s, we employed mice with a conditional deletion of the 75-base pair AU-rich element (ARE) in the 3’ region of the *Csf2* gene. This ARE motif normally destabilizes *Csf2* transcripts to prevent overproduction of GM-CSF, but in *Csf2^fx-ARE^* mice crossed with mice expressing Cre recombinase under control of the *Meox2* gene promoter (*Csf2*^Δ*ARE*^ mice), the ARE motif is deleted during early embryogenesis and *Csf2* transcripts are stabilized with consequent overproduction of GM-CSF ^31^. We confirmed that GM-CSF levels in lung homogenates of *Csf2*^Δ*ARE*^ mice were elevated 2-3 times over those of WT mice and *Csf2^fx^* mice lacking the *Meox2^Cre^* gene (Fig. 4b). Flow cytometric analyses of lung cDCs (Extended Data Fig. 6a) showed that *Csf2*^Δ*ARE*^ mice have significantly more total cDC2s compared with control mice, while having significantly fewer cDC1s (Fig. 4c). This increase in cDC2s was primarily due to an increase of CD301b^+^ cDC2s, as Ly6C^+^ cDC2s were moderately decreased (Fig. 4d), and CD200^+^ cDC2s were unchanged in *Csf2*^Δ*ARE*^ mice at steady state (Fig. 4e). The increase in CD301b^+^ cDC2s was also seen in *Csf2*^Δ*ARE*^ mouse lungs after OVA/HDE inhalation (Fig. 4f).

In agreement with a previous report demonstrating that type 2 alveolar epithelial cells (AT2s) are the major source of GM-CSF ^32, 33^, we confirmed that *Csf2* gene expression is detected in AT2 cells using previously published dataset ^34^ (Extended Data 5c). Consistent with these observations, we found that CD301b^+^ cDC2s reside in the interstitium around alveolar ducts that are surrounded by alveoli ^35^ (Fig. 4g). Together, these results suggest that GM-CSF production by AT2 cells promotes the development or expansion of CD301b^+^ cDC2 within the interstitial space near alveoli and alveolar ducts.

**Fig. 5 |:**
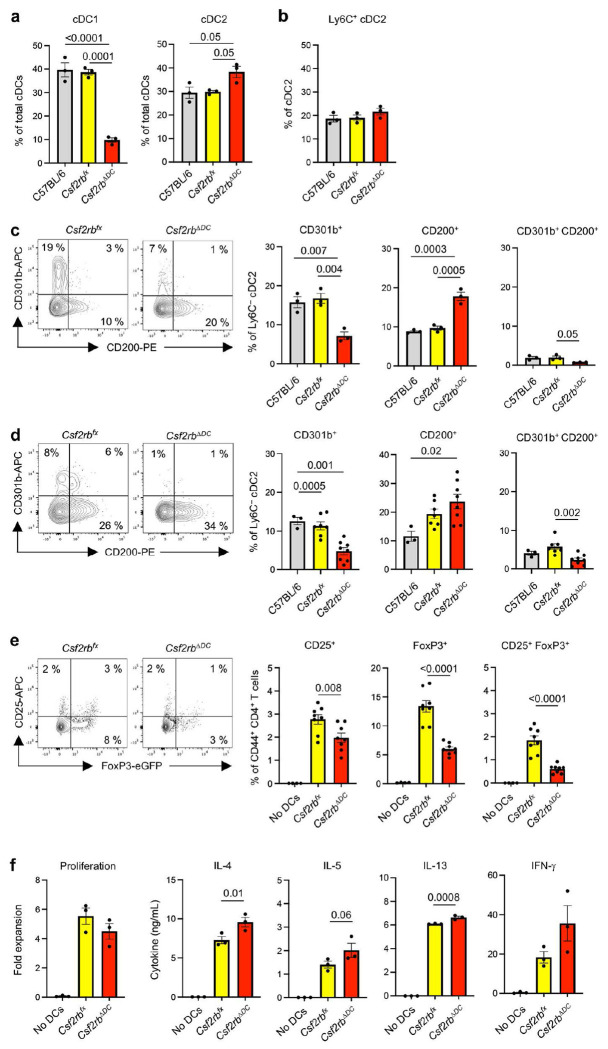
GM-CSF signal is required for CD301b^+^ cDC2 development. **a-d**, Frequencies of cDC1s and cDC2 subsets in mouse lungs at steady state (**a-c**) and after OVA/HDE inhalation (**d**). Each dot represents an individual mouse. Representative results from 2 independent experiments are shown. (**c**, **d**) Representative cytograms (left panels) and compiled data (right panels) of Ly6C^−^ cDC2s are shown. **e**, Treg induction by lung cDC2s. Flow cytometric analysis of CD25^+^ and Foxp3^+^ Tregs after culture of naïve CD4^+^ T cells from *Foxp3^eGFP^* OT-II mice with or without total lung cDC2s (*n*=8). Combined results from two independent experiments are shown. **f**, Effector T cell responses induced by lung cDC2s. Proliferation and cytokine production by T cells, as determined by cell count and ELISA, respectively, after the culture of naïve CD4^+^ T cells from OT-II mice with or without total lung cDC2s (*n*=3). Representative results from two independent experiments are shown. . (**a-f**) Data are presented as mean values ± SEM. *P* values are indicated above the graphs. (**a-d**) Data were analyzed by ordinary one-way ANOVA with Tukey’s multiple comparison test. (**e-f**) Each dot represents separately cultured CD4^+^ T cells. Data were analyzed by one-way ANOVA with Fisher’s LSD test. Comparison between *Csf2rb^fx^* and *Csf2rb*^Δ*DC*^ cDC2s are shown. .

Having established that overproduction of GM-CSF is sufficient to increase numbers of CD301b^+^ cDC2 in the lung, we next tested whether GM-CSF is required for the development of these cells. We employed mice bearing a *Itgax^cre^* transgene and a floxed *Csf2rb* gene encoding the GM-CSF receptor β chain, CSF2RB. In these animals, *Csf2rb* is selectively deleted in *Itgax*-expressing CD11c^+^ cells, which include all lung cDCs (*Csf2rb*^Δ*DC*^). In agreement with previous reports, cDC1s were significantly reduced in *Csf2rb*^Δ*DC*^ mice compared with either WT C57BL/6 mice or *Csf2rb^fx^* control mice lacking the *Itgax^cre^* transgene (Fig. 5a) ^30^. The frequency of total cDC2s at steady state was only slightly increased in *Csf2rb*^Δ*DC*^ mice and no significant differences were seen for Ly6C^+^ cDC2s (Fig. 5b). However, among Ly6C^−^ cDC2s, lung resident CD301b^+^ cDC2s were reduced in *Csf2rb*^Δ*DC*^ mice compared with their WT counterparts (Fig. 5c). Conversely, the frequency of CD200^+^ cDC2s was significantly higher in *Csf2rb*^Δ*DC*^ mice than in control mice. Similar differences between *Csf2rb^fx^* and *Csf2rb*^Δ*DC*^ mice were seen after allergic sensitization with OVA/HDE (Fig. 5d). These results show that GM-CSF is required for the development of CD301b^+^ lung resident cDC2s and for maintaining the normal proportions of CD301b^+^ cDC2s and CD200^+^ cDC2s.

### GM-CSF signaling is required for Treg induction and suppression of allergic inflammation

Our finding that CD301b^+^ cDC2s are decreased in *Csf2rb*^Δ*DC*^ mice suggested that total cDC2s from these animals might have a decreased capacity to induce Tregs. To test this, we purified total cDC2s from *Csf2rb^fx^* and *Csf2rb*^Δ*DC*^ mice and separately cultured them with naïve CD4^+^ T cells prepared from *Foxp3^eGFP^* OT-II reporter mice. cDC2s from *Csf2rb^fx^* control mice readily promoted Treg differentiation, as measured by surface display of CD25 and Foxp3^eGFP^ fluorescence, whereas cDC2s from *Csf2rb*^Δ*DC*^ mice were significantly less potent in this regard (Fig. 5e). Conversely, T cells cultured with cDC2s from *Csf2rb*^Δ*DC*^ mice produced more IL-4, IL-5 and IL-13 than did T cells cocultured with cDC2s from control mice, although the increase in IL-5 did not reach statistical significance (Fig. 5f). Taken together, these data suggest that GM-CSF-dependent cDC2s promote Treg differentiation and suppress Th2 differentiation.

### The role of TGF-β and moderate costimulatory signals in Treg induction by CD301b^+^ cDC2s

**Fig. 6 |:**
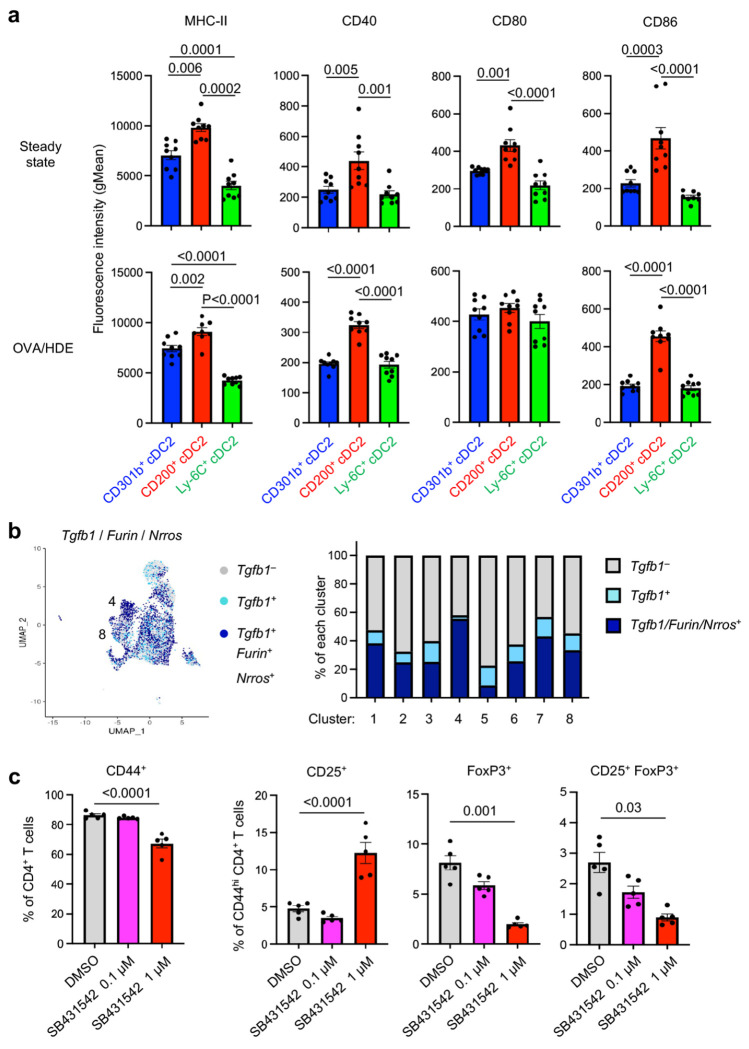
Costimulatory molecule levels on cDC2 subsets and the role of TGF-β on Treg induction. **a**, Surface display levels of MHC-II (I-A) and costimulatory molecules on cDC2 subsets at steady state (top) and 16 h after OVA/HDE inhalation (bottom) were analyzed by flow cytometry. Gating strategy depicted in Extended Data Figure 6b. Data were analyzed by one-way ANOVA with Tukey’s multiple comparison test (*n*=9). Each dot represents an individual mouse. Combined results from two independent experiments are shown. **b**, UMAP of lung cDC2 scRNA-Seq analysis depicting cells expressing *Tgfb1*, *Furin* and *Nrros* (left), and percentage of cells expressing *Tgfb1*, *Furin* and *Nrros* in each cluster (right). **c**, Effect of the TGF-b receptor inhibitor, SB43142, on Treg induction by lung cDC2s. CD25 on and Foxp3^GFP^ in CD4^+^ T cells were analyzed by flow cytometry 5 days after coculture with total lung cDC2s. Gating strategy is depicted in Extended Data Fig. 7a. Each dot represents a separate culture of CD4^+^ T cells. Data were analyzed by one-way ANOVA with Dunnett’s multiple comparison test (**a**), or with Tukey’s multiple comparison test (**c**). Data are presented as mean values ± SEM. *P* values are indicated above the graphs.

Previous studies suggest that high levels of costimulatory signals induce Th2 differentiation *in vitro*, while low level costimulatory signals are associated with Treg induction in skin-draining LNs ^36, 37^. In agreement with those reports, we found that genes encoding the costimulatory molecules CD40, CD80, CD86, as well as ICAM-1, were expressed at lower levels in *Mgl2*^+^ clusters (C4 and C8) compared with *Cd200*^+^ cDC2 clusters (C2 and C5) (Extended Data Fig. 2b). These results prompted us to test display levels of costimulatory molecule proteins on the surface of different cDC2s (Extended Data Fig. 6b). Compared with CD200^+^ cDC2s at steady state, CD301b^+^ cDC2s displayed significantly lower levels of MHC-II, CD40, CD80 and CD86 (Fig. 6a). Similarly, after OVA/HDE inhalation, MHCII, CD40, and CD86 levels were again lower on CD301b^+^ cDC2s than on CD200^+^ cDC2s, while CD80 levels were comparable. These results suggest that low levels of costimulatory molecules might contribute to the Treg-inducing ability of CD301b^+^ cDC2s. However, Ly6C^+^ cDC2s also had relatively low display levels of costimulatory proteins, despite the limited ability of these cDCs to promote Treg differentiation (Fig. 1d). This suggested that CD301b^+^ cDC2s likely have additional features that allow them to promote Treg induction and tolerance.

Immunotolerance is associated with several soluble molecules, including IL-10, IL-35, retinoic acid, and TGF-β ^38^. We therefore investigated the potential contribution of those molecules to Treg induction by CD301b^+^ cDC2s. IL-10 is a well-known immunosuppressive cytokine that decreases secretion of pro-inflammatory mediators from allergen-specific effector T cells ^39, 40, 41^. However, our scRNA-Seq analysis did not reveal selective expression of *Il10* or the IL-10 receptor genes, *Il10ra* and *Il10rb*, in CD301b^+^ cDC2s (Extended Data Fig. 6c). Another cytokine, IL-35, can induce IL-35-producing, inducible Tregs (iTr35) ^42^. *Ebi3* encodes the EBI3 subunit of IL-35, and we found that this gene is expressed by the *Mgl2*^+^ and *Cd200*^+^ cDC2 clusters C2, C4 and C5, with C2 containing the highest expressors (Extended Data Fig. 6c). Another molecule, aldehyde dehydrogenase (ALDH)1A2, can also promote Treg differentiation by generating retinoic acid ^43^, and *Aldh1a2* is expressed by two *Cd200*^+^ cDC2 clusters (C2 and C5). The former cluster C5 is CD301b^−^CD200^+^, while the later cluster C2 is CD301b^+^CD200^+^ (Fig. 2c). Finally, *Tgfb1*, the gene encoding TGF-β, an established inducer of Treg differentiation, is expressed in two *Mgl2*^+^ cDC2 clusters (C4 and C8). Of note, one of these clusters (C4) also expresses genes encoding the TGF-β-activating factors, FURIN and leucine-rich repeat containing (LRRC)33 (LRRC33) (*Nrros*) ^44^ (Fig. 6b, Extended Data Fig. 6d), suggesting that CD301b^+^ cDC2s can produce active forms of TGF-β.

Because CD301b^+^ cDC2 clusters express *Ebi3, Aldh1a2*, and *Tgfb1*, we used selective inhibitors to test the requirement of proteins encoded by those genes in Treg induction. When added to cocultures of cDC2s and naïve CD4^+^ T cells, neither the aldehyde dehydrogenase inhibitor nor the anti-EBI3 neutralizing antibodies affected Treg generation (Extended Data Fig. 7a-c). By contrast, an inhibitor of TGF-β receptor signaling (SB431542) suppressed Foxp3^+^ Treg generation in a dose-dependent manner (Fig. 6c). These results suggest that TGF-β production by CD301b^+^ cDC2s promotes Foxp3^+^ Treg development.

### BM-derived CD301b^+^ cDC2s induce Tregs *in vitro*

**Fig. 7 |:**
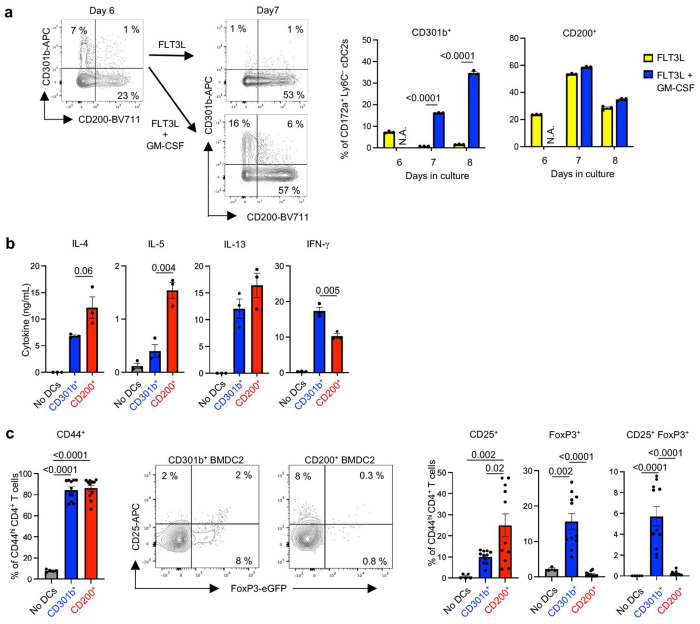
CD301b^+^ BMDC2s induce Tregs *in vitro*. **a**, BMDC2s displaying CD301b and CD200 on their surface. BMDC2s were generated by *in vitro* culture with FLT3L for 6 days, and further cultured with or without GM-CSF. Representative cytograms (left panels) and compiled data (right panels) of flow cytometric analysis are shown. The gating strategy for BMDC2s (CD172a^+^CD11c^+^MHCII^+^CD24^−^Ly6C^−^CD88^−^Live/Dead^−^) is shown in Extended Data Fig. 7d. Data were analyzed by two-way ANOVA with Dunnett’s multiple comparison test. **b**, **c**, Induction of effector CD4^+^ T cells and Tregs by BMDC2 subsets. Cytokine production from OT-II CD4^+^ T cells following culture with the indicated BMDC2 subsets was analyzed by ELISA (*n*=3). Representative data from two independent experiments shown (**b**). Tregs in CD4^+^ T cells from Foxp3^eGFP^ OT-II mice were analyzed by flow cytometry after 5 days culture with BMDC2 subsets (*n*=11). Combined results from two independent experiments shown (**c**). Gating strategies are depicted in Extended Data Fig. 7e and f. Data were analyzed by one-way ANOVA with Tukey’s multiple comparison test. (**a-c**) Each dot represents separately cultured cells. Data are presented as mean values ± SEM. *P* values are indicated above the graphs.

Our findings that CD301b^+^ cDC2s from the lung can induce Treg differentiation *ex vivo* and confer immunotolerance to inhaled allergens *in vivo* suggested that those or similar DCs might have potential as immunotherapeutic agents to suppress allergic disease. However, the lung is not a practical source of immunotherapeutic DCs for humans. We therefore conducted a series of experiments to determine whether tolerogenic cDC2s can be generated from bone marrow. Given that GM-CSF can expand numbers of CD301b^+^ cDC2s in the lung, we tested whether that cytokine can also expand their counterparts in cultures of bone marrow DCs (BMDC2s). We therefore cultured cBMDCs for 6 days in the presence of FLT3L, then added GM-CSF for one day (Fig 7a and Extended Data Fig. 7d). In cultures containing only FLT3L very few immature CD172a^+^CD24^−^ BMDC2s displayed CD301b. However, addition of GM-CSF at day 6 dramatically increased the numbers of those cells. By contrast, numbers of CD200^+^ BMDC2s were unaffected by the presence of GM-CSF. These data show that, as with CD301b^+^ lung cDC2s, GM-CSF also selectively increases numbers of CD301b^+^ BMDC2s.

To test the helper T cell lineage-inducing activity of BMDC2s, CD301b^+^ and CD200^+^ cDC2 subsets were purified by flow cytometry, loaded with OVA_323-339_ peptide, and cocultured with naïve CD4^+^ T cells from OT-II mice. Purified CD200^+^ BMDC2s induced more type 2 cytokine production than did CD301b^+^ BMDCs (Fig. 7b, Extended Data Fig. 7e). However, both BMDC2 subsets activated T cells, as indicated by their increased display of CD44 and CD25. Consistent with lung cDC2s, CD301b^+^ BMDC2s were much more effective at promoting Treg differentiation than were CD200^+^ BMDC2s, as determined by *Foxp3^eGFP^* fluorescence in CD4^+^ T cells (Fig. 7c, Extended Data Fig. 7f). Thus, at least *in vitro*, the highly accessible CD301b^+^ BMDC2s function similarly to their lung counterparts and therefore might have great potential for DC-based immunotherapies to treat inflammatory diseases such as asthma.

## Discussion

Recent research has improved our understanding of the cellular and molecular basis of asthma, but this progress has not led to a corresponding advance in the development of novel and effective therapies. Thus, inhaled corticosteroids remain the primary treatment for asthmatics. Although these powerful anti-inflammatory drugs are effective in the short term, they affect multiple immune cell types and are therefore not appropriate for long term use. Immunotherapies in the form of SCIT and SLIT can be very effective, but they require several years of continued compliance, and this has hindered their ultimate effectiveness. Thus, the development of novel treatments remains critical for improving patient management and for reversing the symptoms of asthma.

One approach to mitigate the symptoms of asthma is to improve the efficiency of immunotherapy so that shorter regimens can be used. It is well established that Tregs suppress allergic responses and their differentiation is promoted *in vitro* by the cytokines TGF-β and IL-2 ^13^, however, how these cells develop *in vivo* is less clear. Our group recently showed that distinct subsets of cDC2s, namely Ly6C^+^ cDC2s and CD200^+^ cDC2s, preferentially promote the differentiation of Th17 and Th2 cells, respectively ^18^. Our current findings reveal that yet another cDC2 subset, which displays the surface protein CD301b, is the dominant cDC2 subset in the lung at steady state conditions, and that this subset preferentially promotes Treg differentiation. This result was unexpected because CD301b^+^ cDC2s in skin-draining LNs can stimulate Th2 differentiation ^45^. Since lung resident cDC2s down-regulate CD301b and upregulate CD200 on their surface upon activation, surface markers of mature cDC2s are likely different between skin and lung.

A role for the IL-10 receptor on cDCs for tolerance induction was previously reported ^40, 46 47^, but our scRNA-Seq analysis did not show strong or selective expression of IL-10 receptor in any of the lung cDC2 clusters. Nonetheless, we cannot exclude the possibility of IL-10 acting on CD301b^+^ cDC2s. We did find that CD301b^+^ cDC2s have lower levels of costimulatory molecules, especially CD86, when compared with CD200^+^ cDC2s. This is in agreement with a recent report that strong costimulatory signals from cDC2s promote Th2 differentiation, whereas moderate costimulatory signals promote Treg differentiation ^48^. Moreover, *Mgl2*^+^ cDC2 clusters, which contain CD301b^+^ cDC2s, express higher levels *Tgfb1* and genes encoding TGF-β-activating factors than do *Cd200*^+^ cDC2s. These genes include *Furin*, which encodes an endoprotease that cleaves newly synthesized, full-length, TGF-β into two peptides: the latency-associated peptide (LAP) and the mature TGF-β cytokine ^49^. CD301b^+^ cDC2s also express *Norros*, which encodes LRRC33, a protein that tethers TGF-β to the cell surface, where it can be activated by αvβ6 and αvβ8 integrins on neighboring cells. Our finding of *Nrros* expression in CD301b^+^ cDC2s is in agreement with its previously reported expression on antigen-presenting cells ^50^. We also found that an inhibitor of TGF-β suppressed Treg induction by cDC2s, providing functional evidence of this pathway. Taken together, our data suggest that weak costimulatory signals and production of mature TGF-β by CD301b^+^ lung cDC2s cooperatively induce Treg differentiation.

Our scRNA-seq data and cDC migration assays revealed that tolerance-inducing CD301b^+^ cDC2s are non-migratory, whereas previous reports have shown that DC migration is required for tolerance induction because mice lacking CCR7 developed airway inflammation even after tolerance induction by OVA inhalation ^51^. However, in that study, mice were sensitized by intraperitoneal (i.p.) injections of OVA/alum prior to challenge with OVA aerosol. It is possible that the requirement for cDC migration in tolerance induction is asthma model-dependent or tissue-specific. Also, *Ccr7*^−/−^ mice and *plt/plt* mice (which lack CCR7 ligands) are known to develop enhanced inflammation ^52, 53^. It is possible, therefore, that the enhanced inflammation previously seen in “tolerized” *Ccr7*^−/−^ mice is due to their enhanced effector response, rather than impaired immunological tolerance. Regardless of the explanation, our data show that Treg development can occur in the lung of *Ccr7*^−/−^ mice, demonstrating that it can proceed in the absence of cDC migration.

GM-CSF drives proliferation of multipotent myeloid progenitors, resulting in the expansion of myeloid cells, including granulocytes, macrophages and cDCs ^54, 55^. Previous studies employing GM-CSF receptor-deficient mice revealed the requirement of GM-CSF signaling for cDC homeostasis in several tissues, including lung. GM-CSF upregulates IRF4 ^33^, a transcription factor required for cDC2 differentiation ^56^. However, the requirement for GM-CSF is not absolute, as some cDC2s are present in lungs of *Csf2r*-deficient mice ^30^. This raises the question of whether some cDC2 subsets are particularly responsive to GM-CSF. In the present study, we found that numbers of lung resident CD301b^+^ cDC2s were significantly reduced in *Csf2rb*^Δ*DC*^ mice, whereas migratory CD200^+^ cDC2s were relatively increased. Conversely, overexpression of GM-CSF in *Csf2*^Δ*ARE*^ mice led to elevated numbers of lung CD301b^+^ cDC2s compared to WT control animals and no change in CD200^+^ cDC2s. These *in vivo* results, together with our finding that GM-CSF expands CD301b^+^ cDC2s derived from BM, demonstrate that GM-CSF promotes expansion of CD301b^+^ cDC2s. Some CD301b^+^ cDC2s remain in lungs of *Csf2rb*^Δ*DC*^ mice, however, suggesting that other growth factors might partly compensate for the absence of GM-CSF. One candidate is M-CSF, as it can partly compensate for FLT3L in *Flt3*-deficient mice ^57^.

The reduction of CD301b^+^ cDC2s in *Csf2rb*^Δ*DC*^ mice was accompanied by a decrease in the ability of their total cDC2s to stimulate Treg differentiation. Paradoxically, however, GM-CSF is reported to enhance immune responses by augmenting the ability of DCs to take up antigen and prime T helper cells. This cytokine also possesses adjuvant activity and can promote allergic airway inflammation and airway hyperresponsiveness ^58 59, 60^. Thus, GM-CSF might enhance the immunostimulatory actions of activated and migratory cDCs (including CD200^+^ cDC2s) while simultaneously promoting immune homeostasis by maintaining the pool of lung resident, Treg-inducing cDCs.

The present study reveals that lung resident cDC2s displaying CD301b are the predominant cDC2 subset at steady state, and that these cDCs preferentially induce Treg differentiation from naïve CD4^+^ T cells. Further, we found that the numbers of these cDC2s are maintained by GM-CSF, and that perturbation of this pathway, through over- or underproduction of that cytokine, changes the balance of Tregs and Th2 cells in a model of allergic asthma. These findings improve our understanding of how lung cDC and Treg development are coordinated *in vivo*. Regarding implications for immunotherapy, our finding that allergen-bearing CD301b^+^ cDC2s confer immunotolerance when adoptively transferred to naïve mice shows that this specific cDC2 subset might have advantages over other cDCs for generating tolerance to inhaled allergen. Further study of these cDCs might also reveal other novel pathways that can be therapeutically targeted to treat or even prevent allergic diseases.

## Methods

### Mice

C57BL/6J (stock 000664), *Cd11c^Cre^* (B6.Cg-Tg*(Itgax-cre)1-1Reiz/*J); stock 008068), CD45.1 (B6.SJL-*Ptprc^a^* *Pepc^b^*/BoyJ; stock 002014), C57BL/6-OT-II TCR transgenic (B6.Cg(TcraTcrb)425Cbn/J; stock 004194), *Foxp3^eGFP^* (B6.Cg-*Foxp3^tm2Tch^*/J; stock 006772), *Ccr7*^−/−^ (C57BL/6-*Ccr7^tm1.1Dnc^*/J; stock 027913), and *Meox2^Cre^* (B6.129S4-*Meox2^tm1(Cre)Sor^*/J; stock 003755) mice were purchased from Jackson Laboratories. CD45.1 OT-II mice were bred by crossing CD45.1 and OT-II mice. *Foxp3^eGFP^* OT-II mice were bred by crossing *Foxp3^eGFP^* and OT-II mice. *Csf2^fx-ARE^* mice were generated by Perry Blackshear (NIEHS) as described previously ^31^. The mice lacking 75 bp AU-rich element (ARE) in *Csf2* mRNA (*Csf2^ΔARE^*) were generated by crossing *Meox2^Cre^* and *Csf2^fx-ARE^* mice ^31^. *Csf2rb^fx^* (C57BL/6-*Csf2rb^tm1c(EUCOMM)Hmgu^*/Orl) mice originally generated by Burkhard Becher (University of Zurich, Switzerland) were obtained from the European Mouse Mutant Archive ^61^. *Cd11c^Cre/wt^ Csf2rb^fx/fx^* (*Csf2rb^ΔDC^*) mice were bred by crossing *Cd11c^Cre^* and *Csf2rb^fx^* mice. Mice were bred and housed in specific pathogen-free conditions at the NIEHS with following housing condition; light cycle: 7AM to 7PM, temperature: 72 ± 2 °F, humidity: 40 to 60 %. Mice were used between 6 and 12 weeks of age. All animal procedures complied with institutional guidelines and were approved by the NIEHS Animal Care and Use Committee.

### Allergic sensitization and mouse model of asthma

For allergic sensitization, mice were lightly anesthetized with isoflurane and given one oropharyngeal (o.p) aspiration of 100 μg LPS-free OVA (Worthington Biomedical) together with 10 μL HDE in a total volume of 50 μL in PBS (OVA/HDE) ^62^. The HDE was prepared as previously described ^23, 63^. Briefly, vacuumed dust samples from homes in North Carolina were passed through a coarse sieve, then extracted at 100 mg/mL with PBS at 4°C with overnight mild agitation. The samples were centrifuged to remove insoluble debris, and supernatants were sterilized by passage through a 0.22-μm filter (Millipore Sigma). Endotoxin concentration was 50 ng LPS/10 μL HDE, as measured by a Limulus Amebocyte Lysate assay (Lonza, catalog #50-648U). For the mouse model of allergic asthma, animals were lightly anesthetized with isoflurane and sensitized with two o.p. aspiration of OVA/HDE 7 days apart. Seven days after the second sensitization, mice were then challenged by exposure to aerosolized 1% OVA in sterile PBS for 1 h. Mice were harvested 48 h post-challenge and bronchoalveolar lavage fluid (BALF) and lung tissue were collected. Lung tissues were incubated in 500 μL complete RPMI1640 containing 10% fetal bovine serum (Hyclone/Cytiva, West Sacramento, CA), penicillin/streptomycin and 50 ng/mL μ-mercaptoethanol (cRPMI-10) supplemented with 10 μg/mL OVA. To induce tolerance, the same procedure was followed except that seven days prior to the first sensitization, 1x10^5^ cDCs were incubated with 10 nM OVA_323-339_ (New England Peptide) *ex vivo* for 1 h, washed, and adoptively transferred by o.p. aspiration.

### Flow cytometric analysis and sorting

Cells were diluted to 1-2x10^6^/100 μL and incubated with a non-specific binding blocking reagent cocktail of anti-mouse CD16/CD32 Ab (2.4G2) (10% culture supernatant), 5% normal mouse and 5% rat serum (Jackson ImmunoResearch) ^64^. Fluorochrome-conjugated antibodies (Abs) against cell surface antigens were obtained from BD Biosciences (BD), BioLegend (BL), R&D Systems (RD), Miltenyi Biotec (MB), or eBioscience/ThermoFisher Scientific (eBio). The Abs included PE-anti-mouse CD3e (145-2C11, BL 100307; 1 μg/mL), BUV395-anti-mouse CD4 (RM4-4, BD 740209; 1 μg/mL), BUV395-anti-mouse CD11b (M1/70, BD 565553; 1 μg/mL), BV510-anti-mouse CD11b (M1/70, BL 101263; 1 μg/mL), PerCP-Cy5.5-anti-mouse CD11c (N418, eBio 45-0114-82; 1 μg/mL), AF488-anti-mouse CD11c (N418, eBio. 53-0114-82; 1 μg/mL), BV510-anti-mouse CD14 (Sa14-2, BL 123323; 1 μg/mL), APC eF780-anti-mouse CD14 (Sa14-2, BL 123331; 1 μg/mL), BUV395-anti-mouse CD24 (M1/69, BD 744471; 1 μg/mL), PE-Dazzle-anti-mouse CD24 (M1/69, BL 101837; 1 μg/mL), APC-anti-mouse CD25 (3C7, BL 101910; 1 μg/mL), FITC-anti-mouse CD40 (3/23, BD 561845; 1 μg/mL), BV510-anti-mouse CD44 (IM7, BL 103044; 1 μg/mL), FITC-anti-mouse CD45 (30-F11, BL 103108; 1 μg/mL), APC-Cy7-anti-mouse CD45.1 (A20, BL 110716; 1 μg/mL), BV510-anti-mouse CD45.2 (104, BL 109837; 1 μg/mL), PerCP-Cy5.5-anti-mouse CD45RB (C363-16A, BL 103313; 1 μg/mL), FITC-anti-mouse CD80 (16-10A1, eBio 11-0801-82; 1 μg/mL), BUV395-anti-mouse CD86 (P03, BD 745716; 1 μg/mL), FITC-anti-mouse CD86 (GL1, BD 561962; 1 μg/mL), PerCP-Cy5.5-anti-mouse CD88 (20/70, BL 135813; 1 μg/mL), PE-anti-mouse CD88 (20/70, BL 135806; 1 μg/mL), APC-anti-mouse CD88 (20/70, BL 135808; 1 μg/mL), BV711-anti-mouse CD88 (20/70, BD 743773; 1 μg/mL), BV510-anti-mouse CD103 (M290, BD 563087; 1 μg/mL), AF700-anti-mouse CD116 (698423, RD FAB6130N; 1 μg/mL), FITC-anti-mouse CD172a (P84, BD 560316; 2.5 μg/mL), BV711-anti-mouse CD200 (OX-90, BD 745548; 1 μg/mL), PE-anti-mouse CD200 (OX-90, BL 123807; 0.5 μg/mL), AF647-anti-mouse CD200 (OX-90, BL 123816; 1 μg/mL), PE-anti-mouse CD301b (URA-1, BL 146804; 1 μg/mL), APC-anti-mouse CD301b (URA-1, BL 146813; 1 μg/mL), BUV737-anti-mouse F4/80 (T45-2342, BD 749283; 1 μg/mL), PE-Dazzle594-anti-mouse F4/80 (BM8, BL 123146; 1 μg/mL), BV711-anti-mouse Ly-6A/E (D7, BL 108131; 0.5 μg/mL), BV510-anti-mouse Ly-6C (HK1.4, BL 128033; 1 μg/mL), FITC-anti-mouse Ly-6C (AL-21, BD 553104; 2.5 μg/mL), APC eFluor 780-anti-mouse Ly-6C (HK1.4, eBio 47-5932-82; 1 μg/mL) eFluor450-anti-mouse MHC class-II I-A^b^ (AF6-120.1, eBio 48-5320-82; 1 μg/mL), FITC-rat IgG2a (R35-95, BD 554688; 1 μg/mL), PE-anti-mouse Siglec-F (E50-2440, BD 552126; 0.5 μg/mL), BV711-anti-Siglec-F (E50-2440, BD 740784), APC-anti-Siglec-F (E50-2440, BD 562680; 1 μg/mL), APC-rat IgG2a (eBR2a, eBio 17-4321-81; 1 μg/mL), BV711-rat IgG2a (RTK2758, BL 400551; 1 μg/mL), PE-rat IgG2a (eBR2a, eBio 12-4321-82; 0.5 μg/mL), PE-rat IgG2b (eB149/10H5, eBio 12-4031-82) (0.5 μg/mL), FITC-rat IgM (R4-22, BD 553942; 2.5 μg/mL). Stained cells were analyzed on LSR-Fortessa flow cytometer (BD Biosciences), and the data analyzed using FACS Diva (BD Bioscience) and Cytobank (Cytobank) or FlowJo (Treestar) software. Only single cells were analyzed or purified, and dead cells stained with eFluor780-conjugated Live/Dead dye (ThermoFisher Scientific) were excluded from analysis. For purification, stained cells were sorted using a cell sorter FACS ARIA-II (BD Biosciences). The gating strategies are depicted in Extended Data Figures.

### Isolation of cDCs from the lung and mLNs

Lungs were harvested from untreated mice or at 6h, 16h or 18h post-sensitization with HDE/OVA. Lungs were perfused by PBS injection into the right ventricle. For cDC preparation, lung tissue or mLNs was minced and digested with Liberase TM (100 μg/mL) (Roche), Collagenase XI (250 μg/mL), Hyaluronidase (1 mg/mL) and DNase I (200 μg/mL) (Sigma Aldrich) for 60 min. or 30 min. at 37 °C, respectively ^65^. EDTA (20 mM final concentration) was added to stop the reaction. The digested tissue was then sieved through a 70 μm nylon strainer (BD Biosciences) to generate a single-cell suspension. To enrich for cDCs, low density cells were collected by gradient centrifugation using 16 % Nycodenz (Accurate Chemical), and then washed with PBS containing 0.5 % bovine serum albumin and 2 mM EDTA. CD11c^+^I-A^+^CD88^lo^F4/80^lo^Siglec-F^lo^Live/Dead^−^ cDCs were purified by flow cytometric sorting.

### Coculture of cDCs and CD4^+^ T cells

To isolate CD4^+^ T cells from skin-draining LNs and spleens, single cell suspensions were passed through a 70 μm strainer and T cells were enriched by gradient centrifugation using Histopaque 1083 (Millipore Sigma). naïve CD4^+^ T cells were purified by AutoMACS (depl025 program, Miltenyi) using streptavidin-conjugated MACS beads (Miltenyi) and a biotinylated antibody cocktail containing the following Abs; anti-mouse CD8α (53-6.7, BD 553029; 0.5 μg/mL), CD8b (53-5.8, BD 553039; 0.5 μg/mL), CD11b (M1/70, BD 553309; 0.5 μg/mL), CD11c (HL3, BD 553800; 0.5 μg/mL), CD16/32 (2.4G2, BD 553143; 0.5 μg/mL), CD19 (6D5, BL 115504, lot B288656; 0.5 μg/mL), CD25 (PC61, BL 102004; 0.5 μg/mL), CD44 (IM7, BL 103004; 0.05 μg/mL), CD49b (DX5, BD 553856; 0.5 μg/mL), I-A^b^ (AF6.120.1, BL 116404; 0.5 μg/mL) and Ly-6C/G (RB6-8C5, BD 553125; 0.5 μg/mL) ^65^ (BD Bioscience or BioLegend). naïve CD4^+^ T cells (5×10^4^ cells/well) and cDCs (5×10^3^ cells/well) were cocultured for 5 days in 200 μL complete Iscove’s modified Dulbecco’s medium (IMDM) containing 10 % fetal bovine serum (FBS; certified, Invitrogen), 50 μM β-mercaptoethanol, penicillin and streptomycin in a 96-well U-bottom plate (BD Biosciences) in a CO^2^ incubator ^66^. The cells were harvested and washed 5 days after coculture, and viable cells were counted using Luna-FL cell counter (Logos Biosystems). T cells were then incubated (1×10^5^ cells/200 μL/well) for 24 h in a 96-well flat bottom plate coated with antibodies to mouse CD3e (145-2C11, BL 100331; 1 μg/mL) and CD28 (37.51, BL 102116, 1 μg/mL). Cytokines in the supernatant of incubated T cells were measured by ELISA using Multiskan Ascent plate reader with Ascent 2.6 software (Thermo Electron) according to manufacturer’s instruction.

### Bone marrow-derived cDC culture

BM cells were prepared from femurs, tibiae, humeri and sternums of C57BL/6 mice, and red blood cells were lysed with 0.15 M ammonium chloride and 1 mM potassium bicarbonate. BM cells were then cultured in complete RPMI1640 containing 10% fetal bovine serum (Hyclone/Cytiva, West Sacramento, CA), penicillin/streptomycin and 50 ng/mL β-mercaptoethanol (cRPMI-10) supplemented with 100 ng/mL recombinant human FLT3L (rFLT3L) (NIEHS Protein Expression Core) at 2x10^6^/mL for 6 days ^67^. Half of the media was replaced with fresh media on day 3 of culture. The cells were cultured for an additional 2 days with or without with 10 ng/mL rGM-CSF (R&D Systems, Minneapolis, MN) in the presence of rFLT3L. Surface display of CD200 and CD301b on cultured BMDC2s (CD11c^+^I-A^+^CD26^+^CD172a^+^CD24^−^CD88^−^Live/Dead^−^) were analyzed by flow cytometry.

### Coculture of cDCs and CD4^+^ T cells

To isolate CD4^+^ T cells from skin-draining LNs and spleens, single cell suspensions were passed through a 70 μm strainer and T cells were enriched by gradient centrifugation using Histopaque 1083 (Millipore Sigma). naïve CD4^+^ T cells were purified by AutoMACS (depl025 program, Miltenyi) using streptavidin-conjugated MACS beads (Miltenyi) and a biotinylated antibody cocktail containing the following Abs; anti-mouse CD8α (53-6.7, BD 553029; 0.5 μg/mL), CD8b (53-5.8, BD 553039; 0.5 μg/mL), CD11b (M1/70, BD 553309; 0.5 μg/mL), CD11c (HL3, BD 553800; 0.5 μg/mL), CD16/32 (2.4G2, BD 553143; 0.5 μg/mL), CD19 (6D5, BL 115504, lot B288656; 0.5 μg/mL), CD25 (PC61, BL 102004; 0.5 μg/mL), CD44 (IM7, BL 103004; 0.05 μg/mL), CD49b (DX5, BD 553856; 0.5 μg/mL), I-A^b^ (AF6.120.1, BL 116404; 0.5 μg/mL) and Ly-6C/G (RB6-8C5, BD 553125; 0.5 μg/mL) ^65^ (BD Bioscience or BioLegend). naïve CD4^+^ T cells (5×10^4^ cells/well) and cDCs (5×10^3^ cells/well) were cocultured for 5 days in 200 μL complete Iscove’s modified Dulbecco’s medium (IMDM) containing 10 % fetal bovine serum (FBS; certified, Invitrogen), 50 μM β-mercaptoethanol, penicillin and streptomycin in a 96-well U-bottom plate (BD Biosciences) in a CO^2^ incubator ^66^. The cells were harvested and washed 5 days after coculture, and viable cells were counted using Luna-FL cell counter (Logos Biosystems). T cells were then incubated (1×10^5^ cells/200 μL/well) for 24 h in a 96-well flat bottom plate coated with antibodies to mouse CD3e (145-2C11, BL 100331; 1 μg/mL) and CD28 (37.51, BL 102116, 1 μg/mL). Cytokines in the supernatant of incubated T cells were measured by ELISA using Multiskan Ascent plate reader with Ascent 2.6 software (Thermo Electron) according to manufacturer’s instruction.

### *In vivo* cDC maturation assay

CD200^−^CD301b^−^Ly6C^+^ cDC2s or CD200^−^CD301b^+^Ly6C^−^ cDC2s from the lungs of OVA/HDE-sensitized C57BL/6 mice (CD45.2) were purified by flow cytometry and adoptively transferred into syngeneic CD45.1 mice by o.p. aspiration (0.5-1.5×10^5^ cells/recipient). CD45.2^+^ donor-derived cDC2s in the recipient lungs were analyzed by flow cytometry.

### Lung cDC migration assay

Mice were given PKH26 (10 μM) (Sigma) with or without OVA or OVA/HDE by o.p. aspiration (50 μL/mouse). MLNs were harvested 24 h after treatment, and digested for 30 min. Single cells were stained with Abs after RBC lysis. PKH^+^ migratory cDC2s were analyzed by flow cytometry.

### Analysis of Treg generation in the lung

Naïve CD4^+^ T cells were purified from CD45.1 OT-II mice as described above, and 10^6^ cells were intravenously injected through tail veins. Some recipient mice were then given OVA alone (100 μg) by o.p. aspiration. Lungs of recipient mice were harvested 5 days later, and digested for 30 min. T cells were enriched by gradient centrifugation using Histopaque 1083. Surface proteins and intracellular Foxp3 (FJK-16s, eBio 48-5773-82, 4 μg/mL) were stained with Abs. CD45.1^+^ donor CD4^+^ T cells were analyzed by for flow cytometry.

### Imaging of precision cut lung slices (PCLS)

PCLSs were generated, stained, and visualized as previously described ^68^. Briefly, slices of the right superior lobe of fresh lungs were made using a precision-cut tissue slicer VF-300 Compresstome (Precisionary Instruments) at 110 μm thickness without perfusion. The slices were stained with AF488-anti-CD324 (DECMA-1, BD 560061, 1 μg/mL), PE-anti-CD301b (URA-1, BL 146804; 1 μg/mL) and BV421-anti-CD11c (N418, BL 117343; 1 μg/mL). Stained slices were fixed then analyzed using a multi-photon laser-scanning microscope Zeiss 980 (Carl Zeiss) and Zen software (Bitplane).

### Cellular indexing of transcriptomes and epitopes sequencing (CITE-Seq)

CD11b^+^ cDC2s from HDE/OVA-treated or untreated C57BL/6 mice were purified by flow cytometry and stained with Total A-Seq oligo-conjugated Abs against CD200 (OX-90, BL 123811), CD301b (URA-1, BL 146817) and Ly-6C (HK1.4, BL 128047) according to the manufacturer’s instruction (BioLegend) (https://www.biolegend.com/en-us/totalseq) ^69^. The cells were counted and examined for viability using a TC-20 cell counter (Bio-Rad). Approximately 10,000 live cells at or above 3×10^5^ cells/mL with 90% or higher viability were loaded into the Single Cell Chip followed by forming single cell emulsion in Chromium Controller (10x Genomics). The cDNA for antibody derived transcripts (ADT) and gene derived transcripts was generated and amplified according to manufacturer’s instructions (10x Genomics and BioLegend). The Gene Expression library and the ADT Library were prepared using the Chromium single cell 3′ library and gel bead kit v3 (10x Genomics, catalogue #PN-1000073) and additional reagents recommended in the protocol of Total A-seq (BioLegend). The two libraries were mixed at 10:1 molar ratio (Gene Expression library to ADT library) and sequenced by the NIEHS Epigenomics and DNA Sequencing Core Laboratory on NovaSeq 6000 (Illumina) with paired-end sequencing. The data were processed using RTA version 2.4.11. A total of 2.4x10^9^ reads were obtained.

### Analysis of CITE-seq data

CITE-Seq raw data processing: Alignment, barcode assignment and unique molecular identifier (UMI) counting Alignment, barcode assignment and unique molecular identifier (UMI) counting was performed using Cell Ranger 3.1.0 and the “cellranger count” command. Alignment was performed with the mouse mm10-1.2.0 reference. The following feature libraries were included for antibody sequencing: CD200 (TCAATTCCGGTAGTC), CD301b (CTTGCCTTGCGATTT), CD11b (TGAAGGCTCATTTGT) and Ly6C (AAGTCGTGAGGCATG). Outputs from filtered count matrices were used for subsequent analyses. Rounded estimates from Cell Ranger were 3,500/5,000/6,500 cells, 195,000/120,000/90,000 mean reads per cells, and 3,000/3,100/3,500 median genes per cell for the 0h, 6h, and 18h samples respectively. 96% of RNA reads mapped to the reference genome and 97% of all barcodes were valid.

scRNA-Seq dimensionality reduction and clustering: Data from scRNA-Seq were processed using the Seurat v3.0 package in R version 3.6.1 (http://satijalab.org/seurat/)^70^. Data were filtered on characteristics for homogeneity, including number of features (high threshold: 6000; low threshold: 1000), total RNA counts (high threshold: 50,000; low threshold: 250), proportion cycling (high threshold: 0.08; low threshold: 0.02), and proportion of mitochondrial RNA (high threshold: 0.075; low threshold: 0.005). Data were normalized and scaled for number of RNA features, proportion cycling, and proportion of mitochondrial RNA. Normalized and scaled gene expression data were projected onto principal components (PCs). The first 30 PCs were used for non-linear dimensionality reduction using Uniform Manifold Approximation and Projection (UMAP)^71^. Gene expression and meta data were visualized using this UMAP projection. Clustering was performed using the “FindNeighbors” (k.param = 50), followed by the “FindClusters” (resolution = 0.5) functions of the Seurat v3.0 package in R version 3.6.2^70^. Cluster marker genes from res.0.5 in Seurat (described above) were generated by the “FindAllMarkers” function. Spliced and unspliced counts matrices were constructed with the velocyto “run10x” function ^28^, followed by RNA velocity analysis via scVelo version 0.2.4 ^72^, according to the authors’ tutorials at https://scvelo.readthedocs.io/en/stable/. The results are displayed as velocity stream and latent time views with scVelo’s dynamical mode. Expression of mouse *Csf2* gene in the lung was analyzed using a scRNA-Seq dataset previously published by Han *et al.*^34^, and visualized by UMAP projection.

### Statistics

Data are presented as mean ± SEM. Statistics to analyze differences among groups using Prism software are indicated in figure legends. Outliers identified by GraphPad Prism ROUT method (Q=1%) were removed from analysis. *P*<0.05 was considered significant.

## Supplementary Material

1

## Data Availability

CITE-Seq data of CD11b^+^ cDC2s have been deposited in GEO under accession code GSE261034. All other data supporting the findings of this study are available from the corresponding author upon request. The raw data used for generating graphs presented in this manuscript are provided in a Source Data file.
