# Supplementary material for: GM-CSF-dependent CD301b+ lung dendritic cells confer tolerance to inhaled allergens
Source: Res Sq. 2024 Jun 4:rs.3.rs-4414130. Preprint. [Version 1] doi: 10.21203/rs.3.rs-4414130/v1 (PMC11177951; doi:10.21203/rs.3.rs-4414130/v1)
Supplement: 1 [file NIHPPrs4414130v1-supplement-1.pdf]

Extended Data Figure legends

Extended Data Fig. 1 | cDC2 subset sorting and T cell stimulation. A, Gating strategy for sorting of cDC1s and cDC2 subsets. cDC1s: CD11c^{+}I-A^{+}CD11b^{-}CD103^{+}CD88^{-}SiglecF^{-}F4/80^{-}. cDC2s: CD11c^{+}I-A^{+}CD11b^{+}CD103^{-}CD88^{-}SiglecF^{-}F4/80^{-}. The gating strategy was used for Fig. 1c and d. **b**, Gating strategy for Treg analysis used for Fig. 1c and d. Tregs were CD4^{+}CD3ε^{+}CD44^{+}I-A^{-}Live/Dead^{-}. **c**, Gating strategy for cDC2 subset purification used for Extended Data Fig. 1d. **d**, Proliferation of and cytokine production from CD4^{+} T cells following 5 days culture of naïve CD4^{+} T cells from OT-II mice with the indicated lung cDC2 subsets purified from OVA/HDE-inhaled C57BL/6 mice. Data are presented as mean values ± SEM. Each dot represents separately cultured CD4^{+} T cells. Data were analyzed by one-way ANOVA with Tukey's multiple comparison test (n=3). P values are indicated above the graphs. Representative results from 2 independent experiments are shown.

Extended Data Fig. 2 | scRNA-Seq analysis of cDC2s. a, Gating strategy for cDC2 subset sorting for scRNA-sequencing (Fig. 2a-e). **b**, Expression of genes encoding proteins for antigen presentation and costimulation displayed on UMAPs. **c**, Violin plot of Ly6C protein levels on cDC2 clusters analyzed by CITE-Seq. **d**, RNA velocity analysis using stream view (dynamic mode) representing RNA directionality at each time point and combination.

Extended Data Fig. 3 | Gating strategies for cDC2 analysis and adoptive transfer of Ly6C^{+} cDC2s. **a**, Gating strategy for flow cytometric analysis of cDC1 and cDC2 subsets used for Fig. 2f and g. **b**, Gating strategy for CD301b^{+} cDC2 sorting for adoptive transfer in Fig. 2h and Extended Data Fig. 3c. **c**, Adoptive transfer of purified Ly6C^{+} lung cDC2s from C57BL/6 mice to

CD45.1 recipient mice. Representative cytograms of purified donor Ly6C$^+$ cDC2s are shown. **d**, Gating strategy for flow cytometry analysis of donor cDC2-derived cells, and cytograms depicting the phenotype of CD45.2$^+$ donor-derived Ly6C$^+$ cDC2s at 1 day post transfer. Representative results of from 2 independent experiments are shown.

Extended Data Fig. 4 | Gating strategy for flow cytometric analyses of cDC2s and Tregs. **a**, Gating strategy for analysis of migratory cDC2 in mLNs. Phenotype of PKH26$^+$ cells labeled in the lung by dye instillation were analyzed. This gating strategy was used for Fig. 3a and b. **b**, Gating strategy for Treg analysis used for Fig. 3c.

Extended Data Fig. 5 | Expression of genes encoding colony stimulating factor receptors and GM-CSF. **a**, Expression of *Csf2ra, Csf2rb, Csf2rb2, Csf1r and Csf3r* in lung antigen-presenting cells and BM preDCs at steady state and 16 h after HDE/OVA. Transcripts per million (TPM) of the indicated genes from previously published bulk RNA-Seq data are shown [18]. Each dot represents a biological replicate. **b**, UMAPs of lung cDC2 scRNA-Seq analysis displaying the expression of *Csf2ra, Csf2rb* and *Csf2rb2*. **c**, UMAPs displaying *Csf2* gene expression and lung cell annotation in the analysis of previously published single cell mouse cell atlas data [34].

Extended Data Fig. 6. | Expression of genes associated with Treg induction. **a,** Gating strategy for flow cytometry analysis of lung cDC2s from $Csf2^{\Delta ARE}$ and $Csf2rb^{\Delta DC}$ mice, used for Fig. 4c-f, and Fig. 5a-d. **b**, Gating strategy of flow cytometry analysis of lung cDC2 subsets used for Fig. 6a. **c**, **d**, Violin plots of lung cDC2 scRNA-Seq analysis displaying expression of genes encoding

proteins that are reported to promote Treg induction (**c**), and genes encoding FURIN and LRRC33 that are required to activate TGF-β (**d**).

Extended Data Fig. 7 | The effect of inhibitors on Treg induction and Treg induction by BMDC2s. **a**, Gating strategy for Treg analysis used for Fig. 6c and Extended Fig. 7b and c. **b**, **c**, Effect of the ALDH inhibitor, disulfiram (0.1 μM) (**b**), or anti-EBI3 neutralizing antibodies (clone V1.4C4.22; 10 μg/mL) (**c**) on Treg induction by lung cDC2s. Naïve $CD4^+$ T cells from $Foxp3^{eGFP}$ OT-II mice were cultured with total cDC2s for 5 days and analyzed by flow cytometry. Higher doses of disulfiram were cytotoxic (data not shown). Data are presented as mean values ± SEM. Each dot represents a separate culture of $CD4^+$ T cells. Data were analyzed by two-tailed t-test (n=5). P values are indicated above the graphs. Representative results from 2 independent experiments are shown. **d**, Gating strategy for BMDC2 analysis used for Fig. 7a. **e**, Gating strategy for purifying $CD301b^+$ and $CD200^+$ BMDC2s for naïve $CD4^+$ T cell coculture in Fig. 7b and c. **f**, Gating strategy for flow cytometric analysis of Tregs following *ex vivo* culture used for Fig. 7c.
